# The Editor's Letter-Box

**Published:** 1913-02-01

**Authors:** G. Acton Davis

**Affiliations:** Acting Treasurer. St. Bartholomew's Hospital


To Miss Margaret Breay.

Madam,—In reply to your letter of the 20th instant I beg to say that the resolution passed by the committee of this hospital on the 5th ultimo does not, either in intention or effect, deprive the members of the nursing staff of freedom of speech on the question of the State registration of nurses or on any other subject.

The committee's decision is merely that meetings for the discussion of controversial subjects cannot be held in the hospital. The governors have never desired to influence or control the opinions of those in their service, who are absolutely free to hold any views they like; and, outside the hospital, to hold or attend meetings for the discussion of this or any other subject.—I am, Madam, yours faithfully,

(Signed) G. Acton Davis,
Acting Treasurer.

St. Bartholomew's Hospital,
January 23.

